# Cognitive profiles in primary progressive aphasia and its variants

**DOI:** 10.1002/alz70857_099014

**Published:** 2025-12-24

**Authors:** Lucia Fernandez‐Romero, Silvia Oliver‐Mas, Cristina Delgado‐Alonso, María Díez‐Cirarda, Esther Valiente‐Gordillo, Juan Ignacio López‐Carbonero, Jorge Matias‐Guiu, María José Gil‐Moreno, Jordi A Matias‐Guiu

**Affiliations:** ^1^ Hospital Clinico San Carlos, Madrid, Madrid, Spain; ^2^ Hospital Clínico San Carlos, Madrid, Madrid, Spain; ^3^ Hospital Clinico Universitario San Carlos, Madrid, Madrid, Spain

## Abstract

**Background:**

Primary progressive aphasia (PPA) is a neurodegenerative syndrome characterized by the progressive impairment of language abilities. However, recent findings suggest that other cognitive domains, beyond language, may also be affected in the early stages of the disease. Exploring cognitive domains could facilitate the diagnosis of PPA and its classification into the three main variants (non‐fluent (nfvPPA), semantic (svPPA) and logopenic (lvPPA)), which remains challenging. This study aimed to identify non‐linguistic cognitive alterations in PPA and its variants.

**Method:**

A cross‐sectional study including 157 patients with PPA and 74 cognitively unimpaired controls. Patients were classified into nfvPPA (*N* = 58), svPPA (*N* = 19), and lvPPA (*N* = 80) using a language battery, FDG‐PET, and CSF biomarkers. The mean age was 69,97 ± 8.12 years, and the 61.04% were females. Four cognitive domains (attention and working memory, executive functions, episodic memory and visuospatial abilities) were assessed using the ACE‐III and the following cognitive tests: Digit Span, TMT‐A and B, Rey‐Osterrieth Complex Figure (copy and 3‐minute recall), VOSP and Tower of London.

**Result:**

All PPA variants scored significantly worse than the control group across most explored domains. The lvPPA and svPPA groups scored lower on episodic memory compared to the nfvPPA group. The lvPPA group performed worse on visuospatial abilities and executive function than the svPPA group. The nfvPPA group scored lower on attention and working memory tasks compared to the svPPA group.

**Conclusion:**

This study highlights the impairment of non‐linguistic cognitive domains in PPA and its variants, identifying distinct cognitive profiles across variants that may aid in the patient diagnosis, classification, and monitoring of disease progression.

